# Extracellular Histone H3 Induces Pyroptosis During Sepsis and May Act Through NOD2 and VSIG4/NLRP3 Pathways

**DOI:** 10.3389/fcimb.2020.00196

**Published:** 2020-05-05

**Authors:** Chun-xia Shi, Yao Wang, Qian Chen, Fang-zhou Jiao, Mao-hua Pei, Zuo-jiong Gong

**Affiliations:** Department of Infectious Diseases, Renmin Hospital of Wuhan University, Wuhan, China

**Keywords:** histone H3, pyroptosis, sepsis, NOD2, VSIG4

## Abstract

**Background:** Histones could be released from the nucleus when stimulated. Increasing evidence has shown that extracellular histones are associated with a variety of inflammation and diseases. Nucleotide binding oligomerzation domain 2 (NOD2) belongs to the NOD like receptor (NLR) family and is reported to promote apoptosis and aggravate inflammatory response. And V-set and immunoglobulin domain containing 4 (VSIG4), a B7 family-related protein, has been confirmed to mediate transcriptional inhibition of nucleotide-binding oligomerization domain-like receptor protein 3 (NLRP3). However, little is known about the impact of extracellular histones on NOD2 or VSIG4 signal transduction. In this study, we aim to explore the effect and mechanism of extracellular histone H3 on pyroptosis.

**Aim:** The purpose of this work was to investigate the mechanism of extracellular histone H3 on pyroptosis in sepsis.

**Methods:** Lipopolysaccharide (LPS) and histone H3 were used to induce sepsis mice model and damage in ANA-1 macrophages. H3 antibody was applied to antagonize the effect of histone H3. NOD2 inhibitor NOD-IN-1 and VSIG4-siRNA were used to investigate the mechanism of histone H3 on pyroptosis. Enzyme linked immune sorbent assay (ELISA) was applied to detect the level of extracellular histone H3. Real-time PCR and Western blotting were employed to detect the key mRNA and protein levels. The pathology of tissues was detected.

**Results:** The level of extracellular histone H3 was increased after LPS stimulation. The mRNA and protein levels of NLRP3, caspase-1, gasdermin D (GSDMD), interleukin (IL)-1β, IL-18 were increased in LPS group, but suppressed by H3 antibody. And the expression of NOD2, receptor-interacting protein 2 (RIP2) was elevated compared with control group. The expression of VSIG4 was inhibited by LPS and suppression of H3 promoted the protein level of VSIG4. H3 antibody alleviated pathological damages in tissues. Furthermore, the mRNA and protein levels of NOD2 in H3 group was higher compared with control group. The mRNA and protein levels of VSIG4 in H3 group was decreased compared with control group, but up-regulated by NOD-IN-1. Besides, the mRNA and protein levels of VSIG4 in NOD-IN-1 + VSIG4-siRNA group was elevated compared with VSIG4-siRNA group.

**Conclusions:** Extracellular histone H3 induced by LPS could cause pyroptosis during sepsis via NOD2 and VSIG4/NLRP3 pathway.

## Introduction

*Sepsis* is a severe life-threatening systemic inflammatory response syndrome with complex pathogenesis, high mortality, which can cause dysfunction of multiple systems and organs in the body (Rhodes et al., 2017). Immune dysfunction runs through the whole development process of sepsis, and the imbalance between pro-inflammatory mediators and anti-inflammatory mediators plays an important role (Liu and Sun, 2019). Moderate immune response can effectively protect the body, while excessive immune activation or immunosuppression can lead to severe organ dysfunction (Gao et al., 2016). Despite rising medical standards, sepsis still lacks effective treatments. Lipopolysaccharide (LPS) is the main stimulus to induce sepsis, which can activate inflammatory cells through multiple pathways such as toll-like receptor 4 (TLR4) and promote the expression of inflammatory factors (Hayashi and Suzuki, 2015; Xie et al., 2018). Sepsis is closely related to the cascade of cytokines and cytokine storms triggered by pathogen-associated molecular patterns (PAMPs) (Cavaillon, 2018).

A growing number of studies have shown that pyroptosis plays an indispensable role in sepsis. Different from necrosis, pyroptosis undergoes membrane blebbing and produces pyrotic bodies before cell membrane rupture, accompanied with pyknosis and chromatin damage (Chen et al., 2016). Pyroptosis is an inflammatory form of programmed cell death mediated by GSDMD, including caspase-1-mediated canonical pathway and caspase-4/5/11-mediated non-canonical pathway. Inflammasome activates caspase-1/4/5/11, which cleave GSDMD to form honeycomb-like pores on the cell membrane, causing cell swelling and eventually lead to cell rupture and death (Ding et al., 2016). Therefore, inflammasome activation is an important event in the pathogenesis of organ dysfunction in sepsis.

It has been reported that extracellular histones can activate nucleotide-binding oligomerization domain-like receptor protein 3 (NLRP3) inflammasome (Allam et al., 2013), but its mechanism in sepsis pyroptosis is incompletely clear. Histones are important structural elements of nuclear chromatin, while extracellular histones are cytotoxic and can cause immune damage (Allam et al., 2014). Histones can directly activate pattern recognition receptors and indirectly induce cell necrosis to produce local cytokines, thus leading to peritonitis (Allam et al., 2013). In acute lung injury (ALI), extracellular histones are important effectors of tissue damage and inflammation (Bosmann et al., 2013). Circulating histone H3 levels in patients with sepsis are associated with mortality and negatively correlated with antithrombin levels and platelet counts (Wildhagen et al., 2015). Therefore, it is of great significance to explore the role and mechanism of extracellular histones in the pathogenesis of sepsis.

Nucleotide binding oligomerzation domain 2 (NOD2) belongs to the NOD like receptor (NLR) family and are capable of interacting with multiple proteins and modulate immune responses in a stimuli-dependent manner. It has been reported that activated NOD2 can recruit receptor-interacting protein 2 (RIP2), and conducts signal through nuclear factor (NF)-κB and MAPK pathway (McDonald et al., 2005; Kanneganti et al., 2007). In myocardial ischemia-reperfusion injury, NOD2 promotes myocardial apoptosis by activating NF-κB signaling pathway, and aggravates inflammatory response (Liu et al., 2016). Besides, NOD2 can exacerbate inflammation and podocyte insulin resistance to promote renal injury in diabetic nephropathy (Du et al., 2013). V-set and immunoglobulin domain containing 4 (VSIG4), also nominated as complement receptor of the Ig superfamily (CRIg) or Ig superfamily protein 39 (Z39Ig), is a B7 family-related protein. VSIG4 has been confirmed to mediate transcriptional inhibition of NLRP3 (Huang et al., 2019). And expression of VSIG4 attenuates hepatic inflammation and fibrosis in high fat diet (HFD)-induced mice (Li et al., 2019). We wonder if NOD2 and/or VSIG4 involve(s) in pyroptosis induced by extracellular histones.

In this study, we adopted LPS to induce sepsis mice model and LPS or H3 to induce damage in ANA-1 cell line. NOD2 inhibitor NOD-IN-1 and VSIG4-siRNA were used as interventions to further investigate the mechanism of histone H3 on pyroptosis in sepsis.

## Materials and Methods

### Chemicals and Reagents

RPMI 1640 medium and fetal bovine serum (FBS) were purchased from Gibco (NY, USA). LPS (purity of 99%, from Escherichia coli O55:B5) was obtained from Sigma (St. Louis, USA, #L2880). Histone H3 was obtained from Roche Life Science (Stockholm, Sweden). H3 neutralizing antibody was obtained from Abcam (Cambridge, MA, USA, #ab1791). NOD-IN-1 (purity of 99%) was obtained from MCE (USA). VSIG4-siRNA was obtained from RiboBio (Guangzhou, China). The antibodies to H3, NLRP3, GSDMD, IL-1β, IL-18 were purchased from Cell Signaling Technology (Boston, USA). GAPDH, NOD2, RIP2 antibodies were obtained from Proteintech (Wuhan, China). VSIG4 antibody was obtained from Bioss (Beijing, China). Caspase-1 antibody was obtained from Santa Cruz Biotechnology (Dallas, Texas, USA). Secondary antibodies applied were the goat anti-rabbit/mouse fluorescent antibody purchased from LI-COR Biosciences, Inc. (Lincoln, USA). RNAiso Plus, PrimeScript™ RT reagent kit and SYBR Premix Ex Taq kit were purchased from TaKaRa (Japan). Histone H3 ELISA Kit was obtained from Bioswamp (Wuhan, China). IL-1β, IL-18 ELISA Kits were purchased from Elabscience (Wuhan, China).

### Cell Culture and Chemical Treatment

RPMI 1640 medium mixed with 10% FBS was used to culture ANA-1 cells in an incubator at 37°C, 5% CO_2_, and saturated humidity. The macrophages were firstly divided into control group and LPS group. The cells were passed in 6-well plates and cultured to 70% density. LPS (10 μg/ml) (Le et al., 1998) was used to stimulate the cells. The supernatants were harvested after 24 h to detect histone H3 levels. Next the cells were divided into control group and H3 group. H3 (50 μg/ml) (Xu et al., 2009) was added into cells. After 12 h, the cells were harvested to detect the expression of related proteins. And then the macrophages were divided into control group, LPS group, LPS + H3 antibody group. LPS was used to stimulate the cells excluding the control group. H3 antibody (10 μg/ml) (Kang et al., 2014) was added into LPS + H3 antibody group 2 h prior to treatment with LPS. The cells were harvested 24 h after the addition of LPS.

### siRNA Transfection

Cells were seeded in 6-well plates at a density of 5 × 10^4^ cells/well to achieve a confluence of 70%. The macrophages were ultimately divided into control group, H3 group, H3 + NOD-IN-1 group, H3 + VSIG4-siRNA group, H3 + NOD-IN-1 + VSIG4-siRNA group. H3 was used to stimulate the cells excluding the control group. VSIG4-siRNA transfection was done 24 h prior to H3 stimulation and NOD-IN-1 was added into medium 2 h prior to H3 stimulation. The cells were harvested 12 h after the addition of H3. The transfection was done using Lipofectamine 2000 (Invitrogen, USA) according to the manufacturer's instructions. The transfection efficiency was confirmed by quantitative Real-Time PCR.

### Immunofluorescence

ANA-1 cells were seeded in a 24-well plate (1.0 × 104 cells/well) and allowed to adhere overnight. After stimulation of LPS for 24 h, cells were fixed with 4% paraformaldehyde for 30 min, permeabilized with 0.2% Triton X-100 (Beyotime, China) for 20 min, blocked with 5% bovine serum albumin (BSA, Beijing Solarbio Science and Technology co., ltd.) for 30 min and incubated with primary antibodies against H3 (1:100) and NOD2 (1:100) at 4°C overnight. The next day, cells were washed and incubated with cy3-labeled fluorescent secondary antibodies (1:100) at room temperature for 1 h. Then the sections were stained with DAPI (Beyotime, China). Observations were performed with a laser scanning confocal microscope (Olympus, Japan, #FV1200).

### Animal Groups

Male C57BL/6 mice (*n* = 18) specific pathogen-free (SPF) (20–25 g) were purchased from Experimental Animal Center of Wuhan University. All animals were acclimatized for 1 week before experimentation and allowed access to food and water freely throughout the acclimatization and experimental periods. They were kept in temperature (22 ± 2°C) with a 12 h light/dark cycle. The mice were randomly divided into three groups: control group, LPS group, LPS + H3 antibody group. Except for control group, all mice were modeled by intraperitoneal injection with LPS (10 mg/kg) (Kong et al., 2018). The control group mice were given an equal volume of saline. H3 antibody (20 mg/kg) (Kang et al., 2014) was given by intraperitoneal injection in LPS + H3 antibody group 2 h before sepsis model protocol. All mice were sacrificed under anesthesia after 24 h when sepsis model established.

### Hematoxylin and Eosin (H&E) and Serum IL-1β, IL-18 Levels Detection

Ten percent neutral-buffered formalin was applied to fix fresh tissue specimens for 2 days. And then tissues were embedded in paraffin, processed for sectioning and staining by H&E. Tissue sections were assessed under BX 51 light microscope (Olympus, Japan). Serum IL-1β, IL-18 levels were detected by ELISA kits according to the manufacturer's instructions.

### Quantitative Real-Time PCR

Total RNAs in ANA-1 cells and tissues were isolated by using RNAiso Plus according to manufacturer's protocol. The cDNAs were produced with a PrimeScript RT reagent kit and incubated at 37°C for 15 min and at 85°C for 5 s. Real-time PCRs were performed using a StepOne Plus device (Applied Biosystems) at 95°C for 10 s, followed by 40 cycles at 95°C for 5 s, and at 60°C for 20 s, according to the instructions for the SYBR Premix Ex Taq kit. The data were analyzed by the 2^−ΔΔCT^ method. All the primers were synthesized by Tsingke (Wuhan, China), and the sequences are listed in Table 1.

**Table 1:** The primer sequence for RT-PCR.

**Gene**		**Primer sequence (5^′^ → 3^′^)**
GAPDH	Forward	CATCACTGCCACCCAGAAGACTG
	Reverse	ATGCCAGTGAGCTTCCCGTTCAG
NLRP3	Forward	TCACAACTCGCCCAAGGAGGAA
	Reverse	AAGAGACCACGGCAGAAGCTAG
GSDMD	Forward	GGTGCTTGACTCTGGAGAACTG
	Reverse	GCTGCTTTGACAGCACCGTTGT
Caspase-1	Forward	GGCACATTTCCAGGACTGACTG
	Reverse	GCAAGACGTGTACGAGTGGTTG
IL-1β	Forward	TGGACCTTCCAGGATGAGGACA
	Reverse	GTTCATCTCGGAGCCTGTAGTG
IL-18	Forward	GACAGCCTGTGTTCGAGGATATG
	Reverse	TGTTCTTACAGGAGAGGGTAGAC
NOD2	Forward	CCTAGCACTGATGCTGGAGAAG
	Reverse	CGGTAGGTGATGCCATTGTTGG
VSIG4	Forward	ATGTGAGGTCACCTGGCAGACT
	Reverse	GCAGGGTTGTAGGTGCTTCAGT

### Western Blotting

Proteins from ANA-1 cells and tissues of mice were separated by sodium dodecyl sulfate polyacrylamide gel electrophoresis (SDS-PAGE) and transferred to a PVDF membrane (Millipore, USA). The membranes were incubated with different primary antibodies and secondary antibody. Finally, expression of each protein was detected by Odyssey infrared imaging system. The dilutions of the primary antibodies were as follows: NLRP3, 1:1,000; GSDMD, 1:1,000; IL-1β, 1:1,000; IL-18, 1:1,000; NOD2, 1:1,000; RIP2, 1:1,000; VSIG4, 1:1,000; caspase-1, 1:500; and GAPDH, 1:2,000. Membranes were also probed for GAPDH as additional loading controls.

### Statistical Analysis

All statistical analyses were performed using SPSS 25.0. The results were presented as mean ± SDs. One-way analysis of variance (ANOVA) or Student's *t*-test was applied to examine the differences between groups. *P* < 0.05 was considered statistically significant.

## Results

### Effect of LPS on Histone H3 in ANA-1 Cells

**Figure 1:**
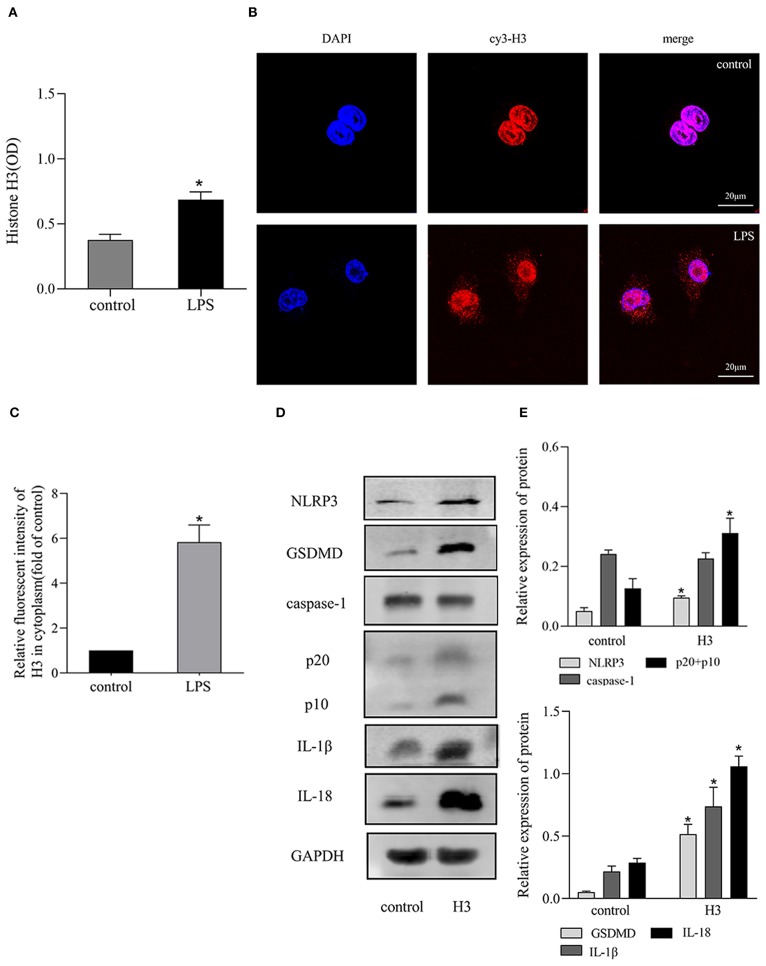
LPS stimulated the release of histone H3 and H3 induced pyroptosis *in vitro*. Cells were stimulated with LPS (10 μg/ml) for 24h or histone H3 (50 μg/ml) for 12 h. **(A)** ELISA results of histone H3 in cell supernatant. **(B,C)** Immunofluorescence was used to detect the expression of histone H3 in cytoplasm. Relative fluorescent intensity of histone H3 in cytoplasm was presented as mean ± SDs of three independent experiments. **(D,E)** Effects of histone H3 on NLRP3, caspase-1, GSDMD, IL-1β, IL-18 protein levels in ANA-1 cells. Odyssey was used to calculate the grayscale of each strip and the results (relative expression to GAPDH) were presented as mean ± SDs based on three repetitions. *P < 0.05, compared with control group.

The expression of histone H3 was increased after LPS stimulation. As shown in Figure 1A, the level of extracellular histone H3 was elevated in LPS group compared with control group (*P* < 0.05). And the expression of cytoplasmic histone H3 was also increased compared with control group (Figures 1B,C).

### Effect of Extracellular Histone H3 on Pyroptosis in ANA-1 Cells

**Figure 2:**
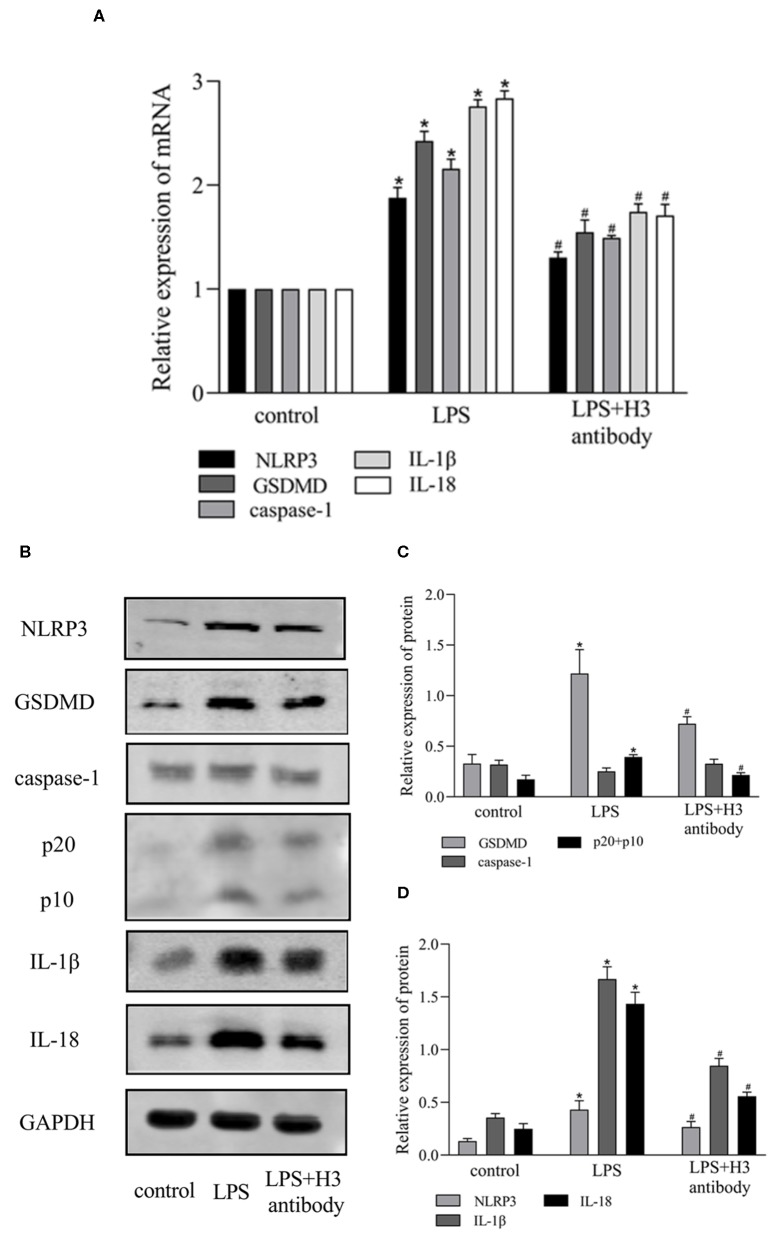
Histone H3 antibody attenuated pyroptosis induced by LPS *in vitro*. LPS (10 μg/ml) was used to stimulate the cells excluding control group. H3 antibody (10 μg/ml) was added into LPS + H3 antibody group 2h prior to LPS. The cells were harvested 24 h after the addition of LPS. **(A)** The mRNA expression (relative to GAPDH) of NLRP3, caspase-1, GSDMD, IL-1β, IL-18 in ANA-1 cells. **(B–D)** The protein levels of NLRP3, caspase-1, GSDMD, IL-1β, IL-18 in ANA-1 cells. Results were presented as mean ± SDs of three repetitions. *P < 0.05, compared with control group; ^#^*P* < 0.05, compared with LPS group.

Extracellular histone H3 could cause pyroptosis in ANA-1 cells. As shown in Figures 1D,E, the levels of pyroptosis-related proteins, NLRP3, caspase-1, GSDMD, IL-1β, IL-18 were significantly increased compared with control group (*P* < 0.05). Likewise, histone H3 antibody could partially block LPS-induced pyroptosis. As shown in Figure 2A, compared with control group, the mRNA levels of NLRP3, caspase-1, GSDMD, IL-1β, IL-18 were higher in LPS group (*P* < 0.05). While H3 antibody suppress the mRNA levels of these cytokines (*P* < 0.05). Similarly, as shown in Figures 2B–D, the western blotting showed that protein levels of NLRP3, caspase-1, GSDMD, IL-1β, IL-18 were increased compared with control group (*P* < 0.05). Whereas, H3 antibody suppress the expression of these proteins (*P* < 0.05). These results confirmed that LPS could induce the release of histone H3, which could cause pyroptosis in ANA-1 macrophages.

### Effect of Histone H3 on NOD2 and VSIG4 Signal Transduction in ANA-1 Cells

**Figure 3:**
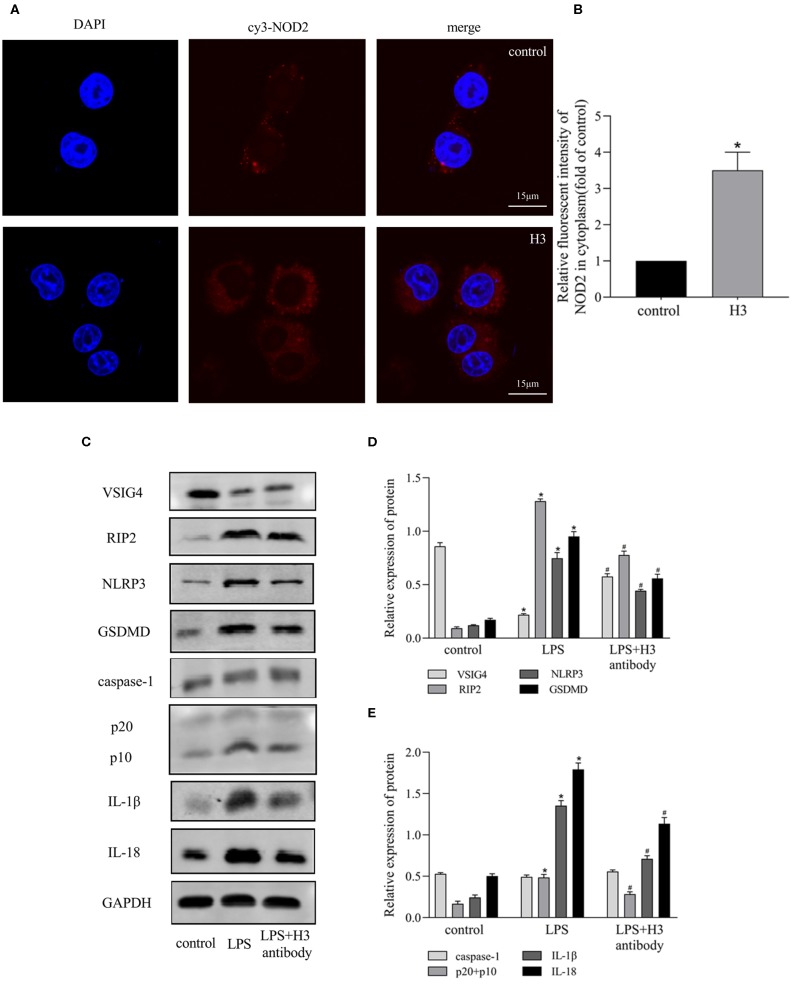
Effects of Histone H3 antibody on LPS-induced NOD2 and VSIG4 signal transduction *in vitro*. Cells were treated the same way as Figure 2. **(A,B)** Immunofluorescence was used to detect the expression of NOD2 in cytoplasm. Relative fluorescent intensity of NOD2 in cytoplasm was presented as mean ± SDs of three independent experiments. **(C–E)** The protein levels of RIP2, VSIG4, NLRP3, caspase-1, GSDMD, IL-1β, IL-18 in ANA-1 cells. Results were presented as mean ± SDs of three repetitions. *P < 0.05, compared with control group; ^#^*P* < 0.05, compared with LPS group.

It has been reported that NOD2 can regulate inflammation and apoptosis (Liu et al., 2016; Panda and Gekara, 2018). And VSIG4 has been confirmed to mediate transcriptional inhibition of NLRP3, macrophages lacking VSIG4 exacerbate pyroptosis in response to NLRP3 inflammasome stimuli (Huang et al., 2019). So we wonder if NOD2 and VSIG4 involve in pyroptosis induced by extracellular histone. As shown in Figures 3A,B, the expression of NOD2 was elevated in H3 group compared with control group (*P* < 0.05). Besides, the protein level of RIP2 was also up-regulated in LPS group (*P* < 0.05) but down-regulated by H3 antibody (*P* < 0.05). However, LPS inhibited the expression of VSIG4 (*P* < 0.05) and suppression of H3 promoted the protein level of VSIG4 (*P* < 0.05) (Figures 3C–E). These results indicated that NOD2 and VSIG4 were involved in pyroptosis.

### The Relationship Between NOD2 and VSIG4 in ANA-1 Cells

**Figure 4:**
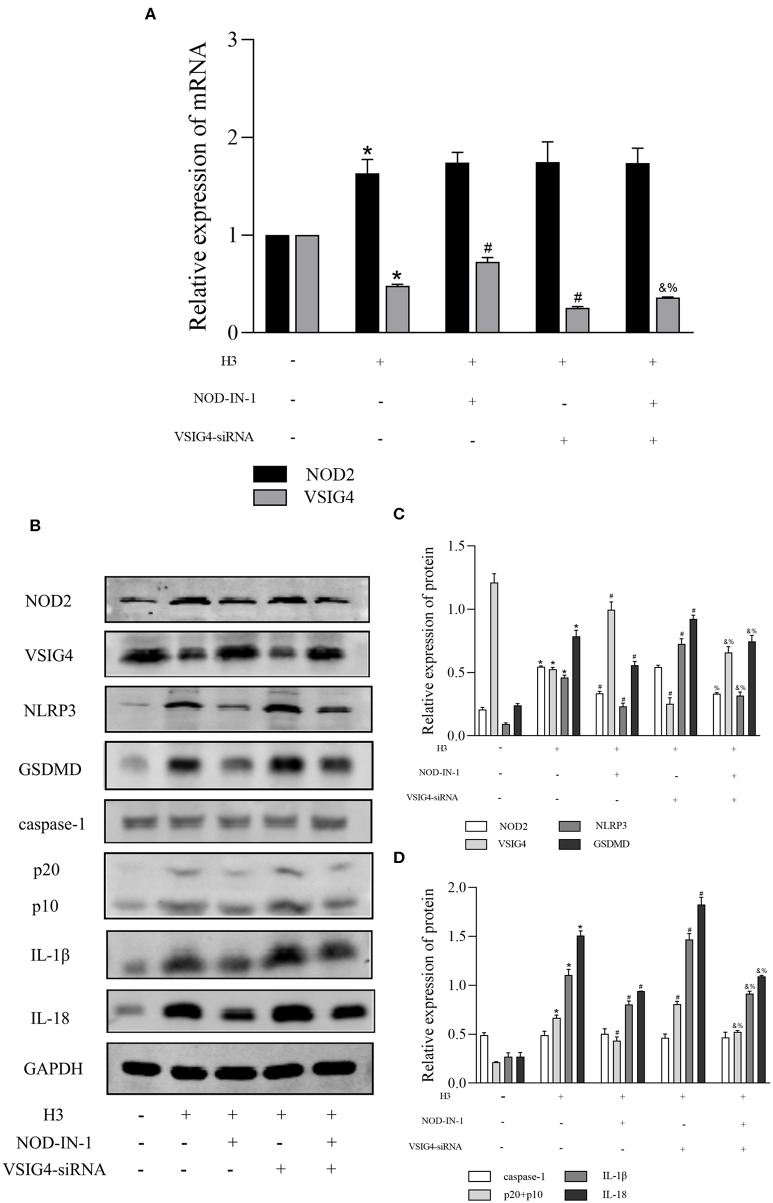
The relationship between NOD2 and VSIG4 and the effect of NOD-IN-1, VSIG4-siRNA on pyroptosis *in vitro*. Histone H3 was used to stimulate cells excluding the control group. VSIG4-siRNA transfection was done 24 h prior to H3 stimulation and NOD-IN-1 was added into medium 2 h prior to H3 stimulation. The cells were harvested 12 h after the addition of H3. **(A)** The mRNA expression (relative to GAPDH) of NOD2, VSIG4 in each group. **(B–D)** The protein expression of NOD2, VSIG4, NLRP3, caspase-1, GSDMD, IL-1β, IL-18 in each group. Results were presented as mean ± SDs of three repetitions. *P < 0.05, compared with control group; ^#^*P* < 0.05, compared with H3 group; ^&^*P* < 0.05, compared with NOD-IN-1 group; ^%^*P* <0.05, compared with VSIG4-siRNA group.

In order to find out whether there is a connection between NOD2 and VSIG4, we used NOD2 inhibitor NOD-IN-1 and VSIG4-siRNA in histone H3 stimulated ANA-1 macrophages. As shown in Figure 4A, the mRNA level of NOD2 in H3 group was higher compared with control group (*P* < 0.05), but there is no statistical significance between H3 group and VSIG4-siRNA group (*P* > 0.05). Also, the mRNA level of NOD2 in NOD-IN-1 group and NOD-IN-1 + VSIG4-siRNA group had no statistical significance (*P* > 0.05). On the contrary, the mRNA expression of VSIG4 in H3 group was decreased compared with control group (*P* < 0.05), but up-regulated by NOD-IN-1 (*P* < 0.05). Besides, the mRNA level of VSIG4 in NOD-IN-1 + VSIG4-siRNA group was elevated compared with VSIG4-siRNA group (*P* < 0.05). The western blotting showed the similarly results (Figures 4B–D). Pyroptosis was also assessed using NOD-IN-1 and VSIG4-siRNA (Figures 4B–D), the western blotting showed that the protein levels of NLRP3, caspase-1, GSDMD, IL-1β, IL-18 were decreased in NOD-IN-1 group compared with H3 group (*P* < 0.05), while these protein levels were elevated in VSIG4-siRNA group compared with H3 group (*P* < 0.05). These results further confirmed the effect of NOD2 and VSIG4 on pyroptosis.

### Histone H3 Antibody Improved Survival Rate and Alleviated Histological Changes in Mice

**Figure 5:**
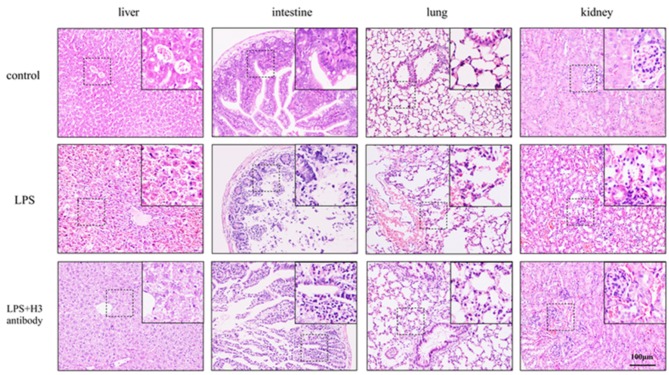
Effect of histone H3 antibody on mice pathological changes of liver, intestine, lung, and kidney.

After 24 h, the mice in control group were all alive, and the survival rate of LPS + H3 antibody group (4 of 6) was significantly higher than that of LPS group (1 of 6). As H&E staining shown in Figure 5, the liver lobule structure in control group was clear and complete, and the liver cell morphology was normal, without necrosis and infiltration of inflammatory cells. In LPS group, the liver tissue structure was seriously destroyed, with extensive necrosis and large amount of inflammatory cells infiltration. While the hepatic lobule structure in LPS + H3 antibody group mice was clearer than that in LPS group, and the infiltration of inflammatory cells and necrotic hepatocytes were also reduced. Besides, the histologic analysis of intestine revealed that intestine mucosal was damaged in LPS group, with denuding and loss of villi, and large reactive lymphoid follicles along with excess of lymphocytes infiltration. However, the damages of intestine were alleviated by H3 antibody. The pathological changes of lung were also assessed. The lung tissues of control group exhibited normal structure. LPS significantly caused lung pathological damage, including edema, pulmonary congestion, neutrophils infiltration, and airway alveolar disarray. Whereas, intervention of H3 antibody inhibited LPS induced lung pathological changes. Moreover, the histological damages of kidney caused by LPS, including mesangial cells proliferation, inflammatory cells infiltration and even necrosis, were alleviated by H3 antibody.

### Histone H3 Antibody Alleviated Pyroptosis Induced by LPS Through NOD2 and VSIG4 Pathways in Mice

**Figure 6:**
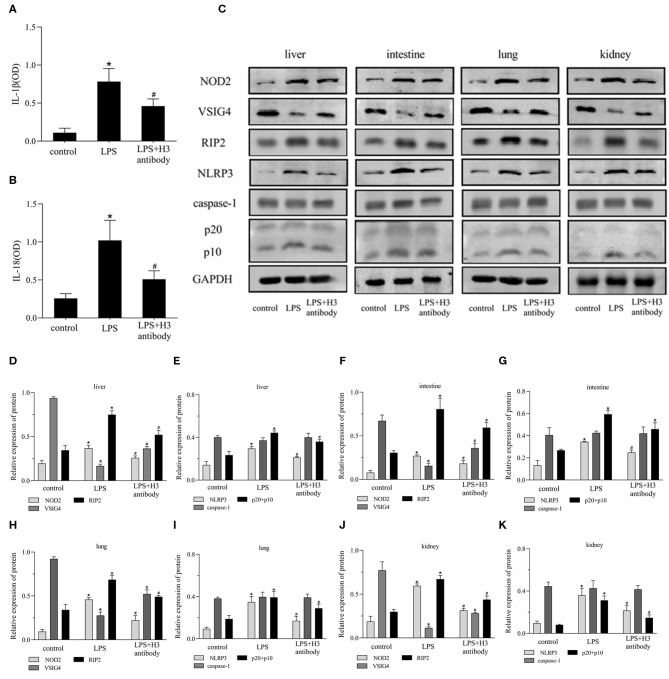
Effect of histone H3 antibody on NOD2, VSIG4, and pyroptosis in mice. All mice were modeled by LPS (10 mg/kg) except for control group. H3 antibody (20 mg/kg) was given in LPS + H3 antibody group 2 h before sepsis model protocol. All mice were sacrificed after 24 h. **(A,B)** ELISA results of IL-1β, IL-18 in mice serum. **(C–K)** Effect of histone H3 antibody on NOD2, RIP2, VSIG4, NLRP3, caspase-1 protein levels in liver, intestine, lung, and kidney. Results were presented as mean ± SDs of three repetitions. *P < 0.05, compared with control group; ^#^*P* < 0.05, compared with LPS group.

As shown in Figures 6A,B, the serum expression of IL-1β, IL-18 in LPS group was significantly increased compared with control group (*P* < 0.05), but decreased by H3 antibody (*P* < 0.05). The protein levels of NOD2, RIP2, NLRP3, caspase-1 were visibly up-regulated and VSIG4 was decreased in liver, intestine, lung and kidney stimulated by LPS (*P* < 0.05). Whereas, H3 antibody reduced the protein levels of NOD2, RIP2, NLRP3, caspase-1, and up-regulated the protein level of VSIG4 (*P* < 0.05) (Figures 6C–K).

## Discussion

*Sepsis* is a major cause of deaths among hospitalized patients worldwide, and the disease progresses rapidly. Despite the current excellent clinical treatments and care measures, the incidence and mortality of sepsis remain high. LPS is the main stimulant of sepsis, which has extensive biological activities and can activate monocytes, macrophages, causing the release of a variety of inflammatory mediators, leading to pathological physiological changes (Cinel and Opal, 2009). The pathophysiological mechanism of sepsis is very complex, including inflammation, immune and coagulation dysfunction and other aspects, involving changes in cell function, metabolism, and microcirculation. In the early stage, sepsis is mainly manifested as excessive inflammatory response. Cytokines, immune system and coagulation system dysfunction lead to vascular endothelial permeability increased, tissue edema, and finally microthrombus, shock, and organ failure. In late period, the main manifestation is excessive immune suppression, which seriously declines the body's resistance and immunity, and finally causes the body to suffer more serious damages (Anas et al., 2010).

The role of pyroptosis activated by inflammasome in sepsis has been reported (Guo et al., 2015; Kim et al., 2016). In sepsis, pyroptosis is necessary to f resist bacterial infection, but excessive pyroptosis can aggravate the inflammatory response. As pathogens and toxins invade the body, they are recognized by pattern recognition receptors, promoting the assembly of inflammasome, activating caspase-1, inducing the maturation and secretion of inflammatory cytokines IL-1β and IL-18, and leading to cell pyroptosis (Kumar, 2018). The activation of inflammasome NLRP3 can lead to massive infiltration of neutrophils and macrophages in liver, kidney, and other organs, and then induce cell pyroptosis (Wu et al., 2015). LPS can also directly bind and activate caspase-11 to induce cell pyroptosis, and simultaneously activate NLRP3 inflammasome, mediate the maturation and secretion of IL-1 and IL-18, and further release damage associated molecular pattern molecules (DAMPs), such as high mobility group box-1 (HMGB1), which have an important impact on the development of sepsis (Shi et al., 2014).

Extracellular histones, which have been reported to activate NLRP3 inflammasome (Allam et al., 2013), are associated with a variety of inflammation and diseases. And it has been reported that neutrophil extracellular traps (NETs), in which histone H3 is a key component, can promote macrophage pyroptosis in sepsis (Chen et al., 2018). Once released from the nucleosome, extracellular histones exert their damaging effects in three ways: (1) induce chemokine release or act as chemokines; (2) by inducing cytokine release and/or apoptosis of adjacent cells and leukocytes; (3) through direct cytotoxicity (Szatmary et al., 2018). Extracellular histones can interact with TLR2, TLR4 (Allam et al., 2012) and TLR9 (Huang et al., 2011) to cause organ damages in acute kidney injury and sterile inflammatory liver injury. Moreover, circulation histones are elevated in acute pancreatitis, and are correlated negatively with leucocyte cell viability (Liu et al., 2017). So we wonder whether histones change in sepsis, and how histones are related to pyroptosis.

In our experiments, LPS was used to stimulate macrophages. The results showed that the level of histone H3 was increased after LPS stimulation. Furthermore, when stimulated with histone H3, pyroptosis-related proteins, NLRP3, caspase-1, GSDMD, IL-1β, IL-18 increased. And cell pyroptosis caused by LPS could be inhibited by H3 antibody. These results indicated that extracellular histone H3 induced by LPS could cause pyroptosis in macrophages. To further explore the mechanisms of histone H3 on pyroptosis, we evaluated the effect of histone H3 on NOD2 and VSIG4 signaling pathways. Our results showed that the expression of NOD2 and RIP2 was elevated compared with control group. However, the expression of VSIG4 was inhibited by LPS and suppression of H3 promoted the protein level of VSIG4. The similar results were also observed in animals. The tissue structures of liver, intestine, lung, and kidney were seriously destroyed by LPS, whereas, inhibition of H3 alleviated the damages induced by LPS. And the protein levels of NOD2, RIP2 were up-regulated in sepsis mice but down-regulated when H3 was suppressed. On contrary, VSIG4 was down-regulated in sepsis mice but up-regulated by H3 antibody. These results revealed that histone H3 could cause pyroptosis through NOD2-RIP2 and VSIG4 signal pathways.

In order to find out whether there was a connection between NOD2 and VSIG4, NOD2 inhibitor and VSIG4-siRNA was used in histone H3 stimulated ANA-1 macrophages. NOD2 and NLRP3 are both involved in pyroptosis, and VSIG4 has been verified to suppress NLRP3 (Huang et al., 2019), our results showed that NOD2 could negatively regulate VSIG4 and positively regulate NLRP3; while VSIG4 had a slight effect on NOD2 and down-regulated NLRP3. These results indicated that NOD2 is a bridge molecule between VSIG4 and NLRP3. Due to limitation of experimental conditions, the specific regulatory relationship between NOD2 and VSIG4 needs further experiments to verify.

In conclusion, extracellular histone H3 induced by LPS could cause pyroptosis during sepsis. The mechanism of extracellular histone H3 on pyroptosis was partly via NOD2 and VSIG4/NLRP3 pathways. The present study not only proved that extracellular histone H3 aggravated inflammation as we previously speculate but also further demonstrated its mechanism on pyroptosis. This experiment provides a new direction of prevention and treatment for sepsis.

## Data Availability Statement

All datasets generated for this study are included in the article/supplementary material.

## Ethics Statement

This animal study was reviewed and approved by Ethics Committee of Renmin Hospital of Wuhan University.

## Author Contributions

ZG contributed to study conceptualization, supervision and reviewed, and edited the manuscript. CS designed the experiments, analyzed the data, and wrote the paper. YW, QC, FJ, and MP performed the experiments and contributed reagents, materials, and analysis tools. All authors approved the final version of the manuscript.

## Conflict of Interest

The authors declare that the research was conducted in the absence of any commercial or financial relationships that could be construed as a potential conflict of interest.
